# Author Correction: Unmodified rabies mRNA vaccine elicits high cross-neutralizing antibody titers and diverse B cell memory responses

**DOI:** 10.1038/s41467-023-39754-1

**Published:** 2023-07-10

**Authors:** Fredrika Hellgren, Alberto Cagigi, Rodrigo Arcoverde Cerveira, Sebastian Ols, Theresa Kern, Ang Lin, Bengt Eriksson, Michael G. Dodds, Edith Jasny, Kim Schwendt, Conrad Freuling, Thomas Müller, Martin Corcoran, Gunilla B. Karlsson Hedestam, Benjamin Petsch, Karin Loré

**Affiliations:** 1 Division of Immunology and Allergy, Department of Medicine Solna, Karolinska Institutet and Karolinska University Hospital, Stockholm, Sweden; 2 Center of Molecular Medicine, Stockholm, Sweden; 3 Astrid Fagraeus Laboratory, Comparative Medicine, Karolinska Institutet, Stockholm, Sweden; 4 Certara USA, Inc, Princeton, NJ USA; 5 CureVac SE, Tübingen, Germany; 6 Institute for Molecular Virology and Cell Biology, Friedrich-Loeffler-Institut, Greifswald-Insel Riems, Greifswald, Germany; 7 Department of Microbiology and Tumor Biology, Karolinska Institutet, Stockholm, Sweden; 8 Present Address: Nykode Therapeutics, Oslo, Norway; 9 Present Address: School of Basic Medicine and Clinical Pharmacy, China Pharmaceutical University, Nanjing, China

**Keywords:** RNA vaccines, Antibodies, Immunological memory

Correction to: *Nature Communications* 10.1038/s41467-023-39421-5, published online 22 June 2023

In this article the affiliation details for Benjamin Petsch were incorrectly given as ‘Certara USA, Inc, Princeton, NJ, USA’ but should have been ‘CureVac SE, Tübingen, Germany’. The original article has been corrected.

