# Supplementary material for: Unmodified rabies mRNA vaccine elicits high cross-neutralizing antibody titers and diverse B cell memory responses
Source: Nat Commun. 2023 Jun 22;14:3713. doi: 10.1038/s41467-023-39421-5 (PMC10287699; doi:10.1038/s41467-023-39421-5)
Supplement: Supplementary file 1 — Supplementary Information [file 41467_2023_39421_MOESM1_ESM.pdf]

Unmodified rabies mRNA vaccine elicits high cross-neutralizing antibody titers and diverse B cell memory responses

Fredrika Hellgren[1,2*]Alberto Cagigi[1,2,3*], Rodrigo Arcoverde Cerveira[1,2*], Sebastian Ols[1,2], Theresa Kern[1,2], Ang Lin[1,2,4], Bengt Eriksson[5], Michael G Dodds[6], Edith Jasny[7], Kim Schwendt[7], Conrad Freuling[8], Thomas Müller[8], Martin Corcoran[9], Gunilla Karlsson Hedestam[9], Benjamin Petsch[6] and Karin Loré[1,2]

[1]Division of Immunology and Allergy, Department of Medicine Solna, Karolinska Institutet and Karolinska University Hospital, Stockholm, Sweden. [2]Center of Molecular Medicine, Stockholm, Sweden. [3]Current affiliation: Nykode Therapeutics, Oslo, Norway. [4]Current affiliation: School of Basic Medicine and Clinical Pharmacy, China Pharmaceutical University, Nanjing, China. [5]Astrid Fagraeus Laboratory, Comparative Medicine, Karolinska Institutet, Stockholm, Sweden. [6]Certara USA, Inc, Princeton, NJ, USA. [7]CureVac SE, Tübingen, Germany. [8]Institute for Molecular Virology and Cell Biology, Friedrich-Loeffler-Institut, Greifswald-Insel Riems, Germany. [9]Department of Microbiology and Tumor Biology, Karolinska Institutet, Stockholm, Sweden.

*** These authors contributed equally**
E-mail corresponding author: karin.lore@ki.se

Supplementary Information:

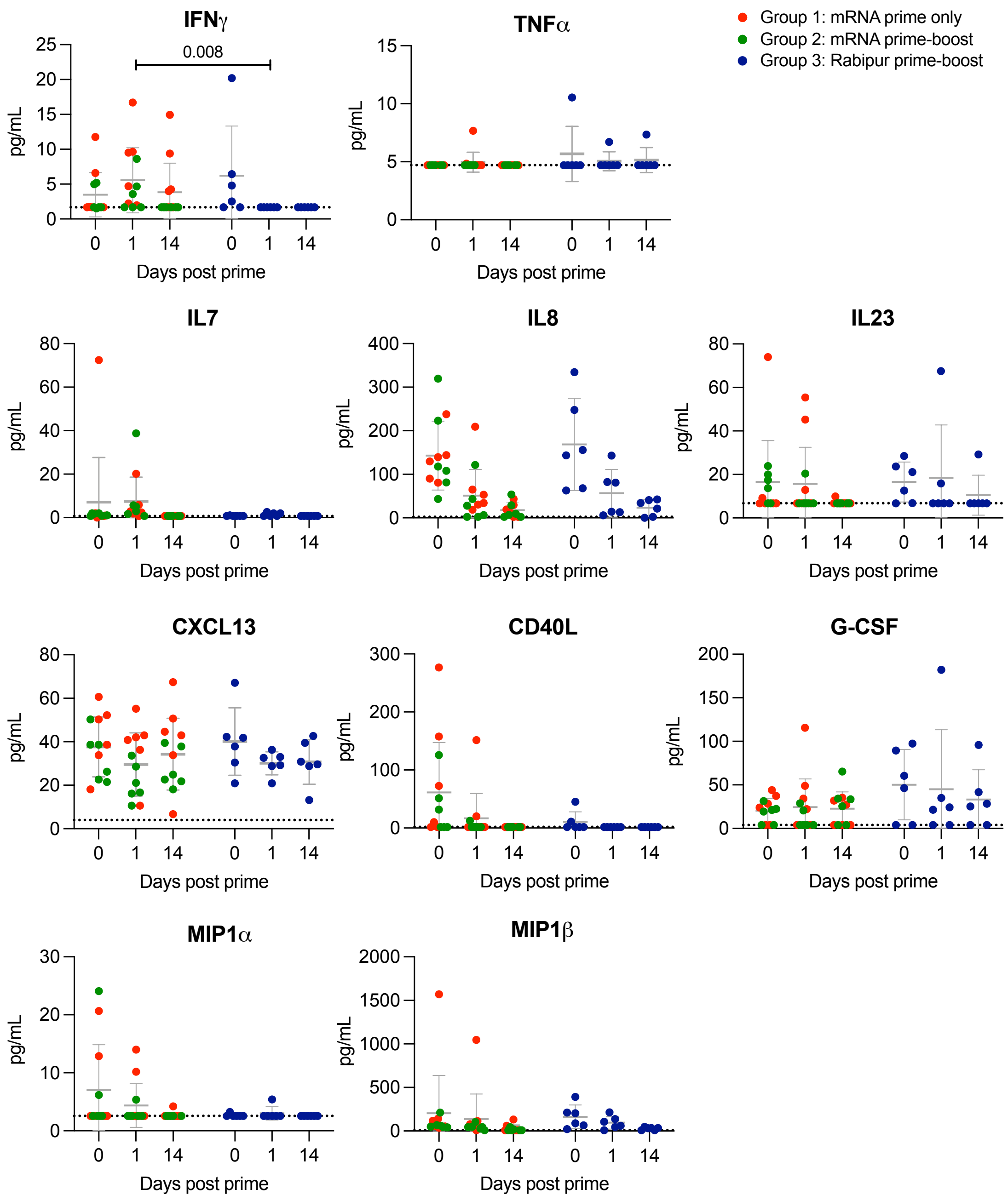

Figure S1

Supplementary Fig. 1: Limited detectable plasma cytokine levels by the mRNA vaccine and Rabipur. Dot plots for each cytokine are shown at the time of vaccination (day 0) and at 24 hours and two weeks after immunization (day 1 and day 14 respectively). Groups 1 and 2 are shown and analyzed as one group, as at this time of the study, they are equivalent having received one dose of the mRNA vaccine. Red, green and blue is the color code assignment for groups 1, 2 and 3 respectively. n=18 biologically independent animals. Cytokine concentrations undetectable or below lower limit of quantitation (LLOQ) are shown as LLOQ value. Dotted lines indicate LLOQ. Statistical differences were assessed at day 1 using Mann-Whitney U test. All statistical tests comparing the study groups were two-tailed tests. Error bars indicate mean ± SD.

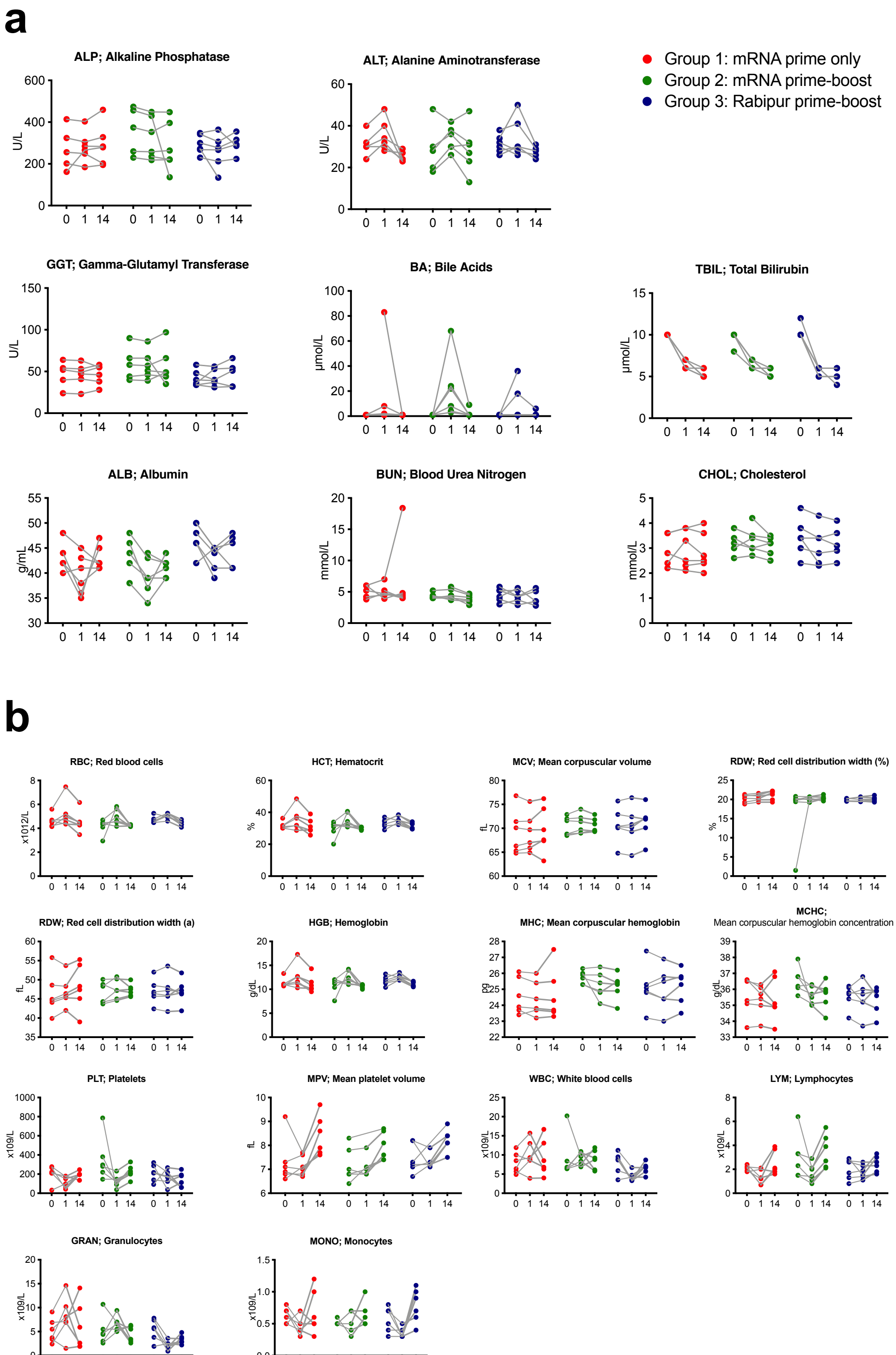

Figure S2

Supplementary Fig. 2: Mild fluctuations in clinical chemistry and complete blood cell count parameters after vaccination. Individual dot plots for **(a)** kidney and liver enzymes and **(b)** blood parameters are shown at the time of vaccination (day 0) and at 24 hours and two weeks after immunization (day 1 and day 14 respectively). Red, green and blue is the color code assignment for groups 1, 2 and 3 respectively. Statistical differences were assessed at day 1 using Kruskal-Wallis test. All statistical tests comparing the study groups were two-tailed tests.

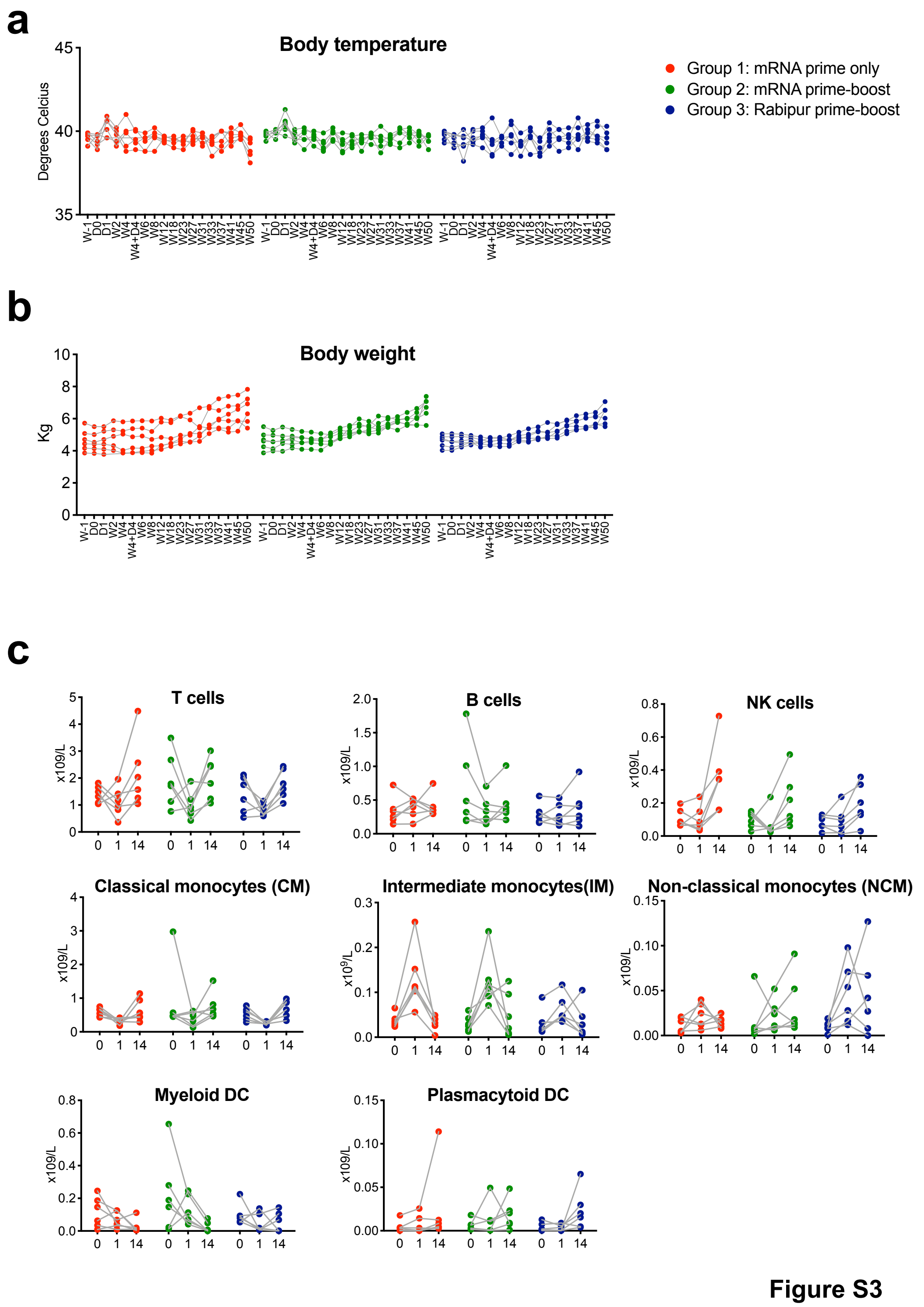

Supplementary Fig. 3: No influence on body weight and temperature and transient effects on immune cell subpopulations after vaccination. Individual dot plots for **(a)** body temperature and **(b)** body weight fluctuations in each group are shown throughout the course of the study. **(c)** Lymphocyte subpopulation fluctuations are shown instead at the time of vaccination (day 0) and at 24 hours and two weeks after immunization (day 1 and day 14 respectively). Red, green and blue is the color code assignment for groups 1, 2 and 3 respectively. Statistical differences were assessed at day 1 using Kruskal-Wallis test. All statistical tests comparing the study groups were two-tailed tests.

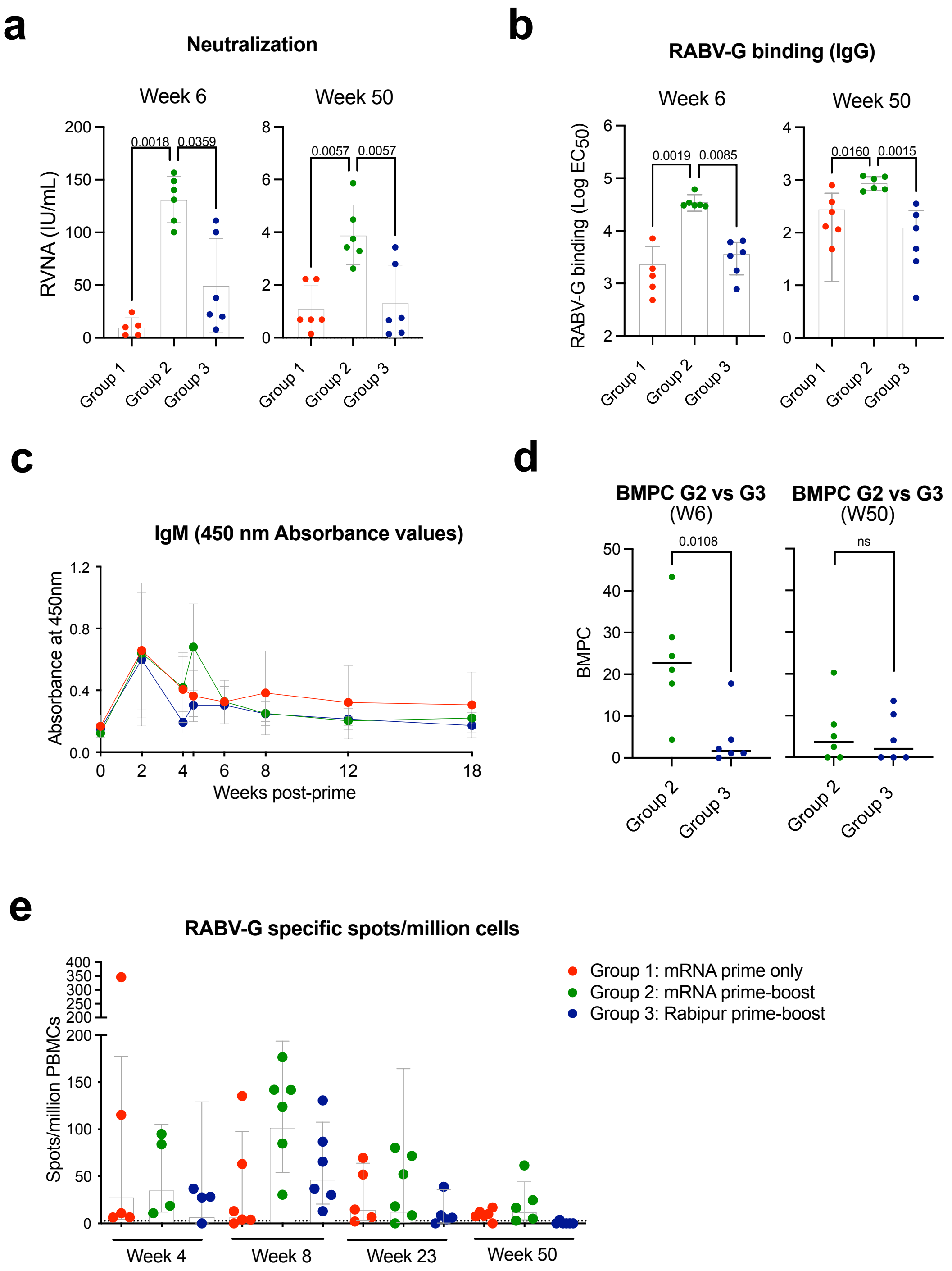

Figure S4

Supplementary Fig. 4: Higher and more durable antibody responses with prime-boost mRNA vaccination. (a) Neutralizing antibody titers comparisons are shown at week 6 (peak responses) and at week 50 (late responses). n=18 biologically independent animals. Statistical differences were calculated using Kruskal-Wallis test. Error bars indicate mean ± SD. **(b)** Total IgG titers comparisons are shown at week 6 (peak responses) and at week 50 (late responses). n=18 biologically independent animals. Statistical differences were calculated using Kruskal-Wallis test. Error bars indicate mean ± SD. **(c)** Total IgM titers measured by ELISA (given the overall low titers, the maximum absorbance at 450nm of a fixed sample dilution was used) are shown up to week 18. The circles represent the single time point average values connected by a line across the different time points measured. n=18 biologically independent animals. Statistical differences were calculated using Kruskal-Wallis test. Error bars indicate mean ± SD. **(d)** Bone marrow plasma cell levels (spots/million cells) for group 2 versus group 3 are shown at week 6 (peak responses) and at week 50 (late responses). n=18 biologically independent animals. Statistical differences were assessed using Mann-Whitney U test. Lines indicate median. **(e)** Longitudinal data of antigen-specific memory B cell derived antibody-secreting cells upon polyclonal stimulation of PBMC enumerated by B cell ELISpot at the different study timepoints. n=18 biologically independent animals. Statistical differences were calculated using Kruskal-Wallis test. All statistical tests comparing the study groups were two-tailed tests. Error bars indicate geometric mean ± geometric SD.

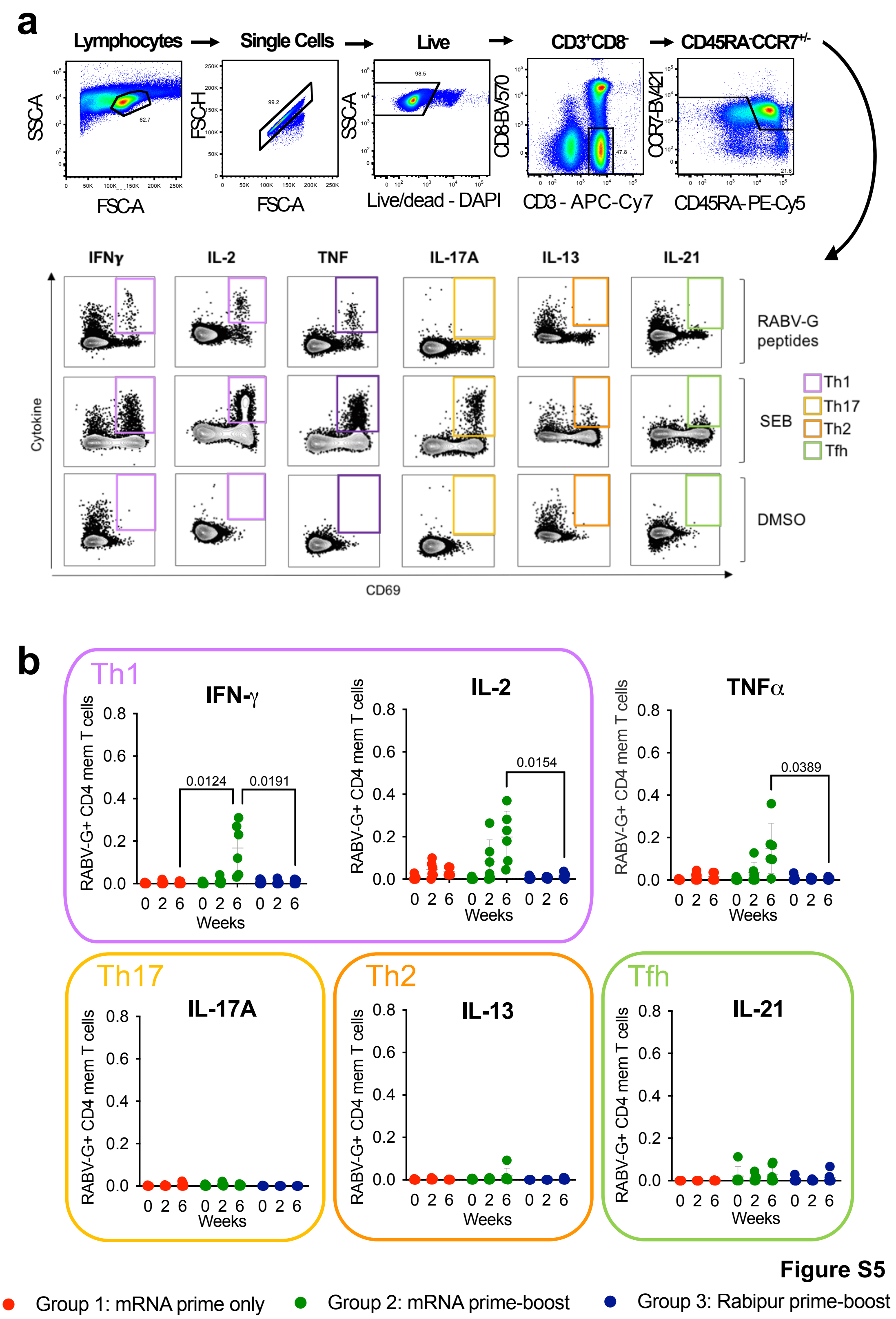

Supplementary Fig. 5: Th1 polarized T cell response induced by mRNA vaccination. (a) Identification of antigen-specific T helper (Th) cell subsets by flow cytometry. Total CD4+ memory Th cells are first identified as live single cells being $CD3^+CD8^-CD45RA^-CCR7^{+/-}$. Within this population, activated cells being $CD69^+$ are analyzed for the presence of different intracellular cytokines. IFNγ and IL-2 identifying the Th1 subset and IL-17A, IL-13 and IL-21 identifying the Th17, Th2 and T follicular helper (Tfh) subsets respectively. TNF was additionally analyzed. For all of the above cytokines, an example is shown without specific stimulation (DMSO), with a general stimulation (SEB) and on cells stimulated with a pool of RABV-G overlapping peptides. **(b)** Longitudinal data showing the level of the aforementioned Th cell subsets prior to immunization (week 0), two weeks after prime (week 2) and two weeks after boost (week 6 from study start). n=18 biologically independent animals. Statistical differences were calculated at weeks 2 and 6 using Kruskal-Wallis test. All statistical tests comparing the study groups were two-tailed tests. All error bars indicate mean ± SD.

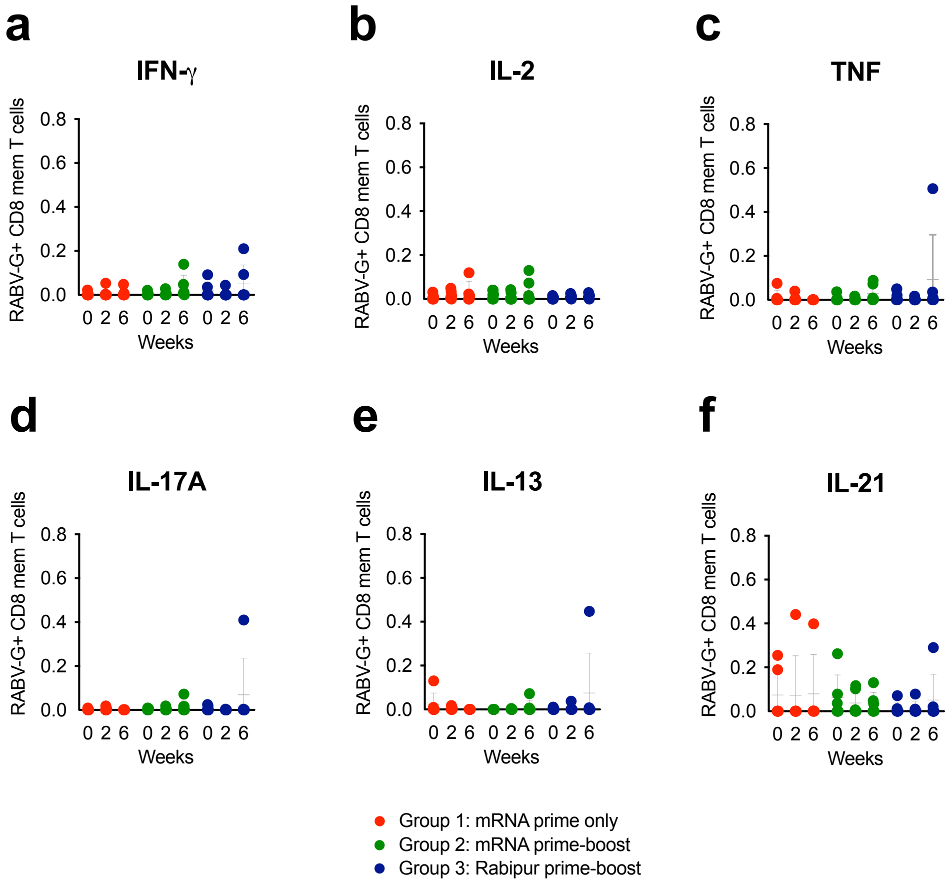

Figure S6

Supplementary Fig. 6: Limited CD8+ T cell responses induced by the vaccines. (a-f) RABV-G specific CD8 T cells. Gated on cytokine-producing CD69+ cells following the same principle as shown for CD4+ T cells in Supplementary Fig. 5a. Showing CD8+ T memory cells expressing CD69 and IFN-y (**a**), IL-2 (**b**), TNF (**c**), IL-17A (**d**), IL-13 (**e**) or IL-21 (**f**) in response to RABV-G overlapping peptide stimulation.Statistical differences were calculated at weeks 2 and 6 using Kruskal-Wallis test. n=18 biologically independent animals. Statistical differences were calculated at weeks 2 and 6 using Kruskal-Wallis test. All statistical tests comparing the study groups were two-tailed tests. Error bars indicate mean ± SD.

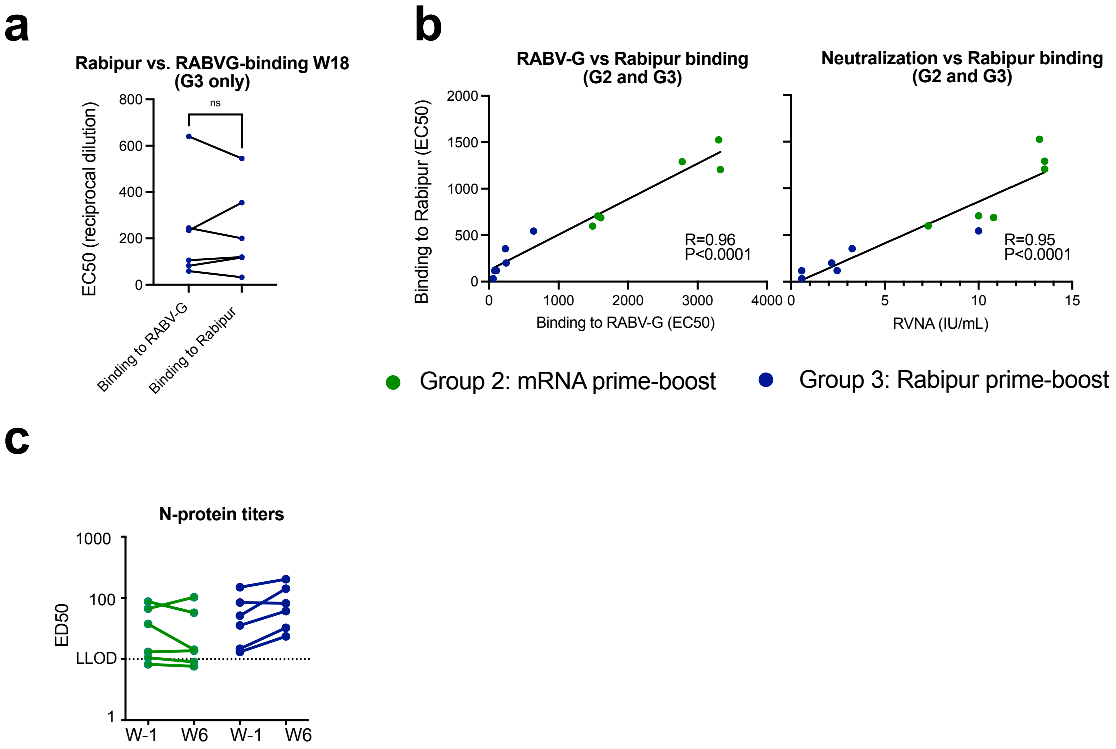

Figure S7

Supplementary Fig. 7: Neutralizing antibodies consist of RABV-G specific antibodies. (a) Binding to Rabipur (EC_{50}) at week 18 is shown for group 3. n=18 biologically independent animals. Statistical differences were assessed using Wilcoxon test. **(b)** Spearman correlation between binding antibody titers to RABV-G and Rabipur and between RVNA and binding titers to Rabipur are shown for groups 2 and 3 also at week 18. **(c)** Rabies nucleocapsid protein binding IgG (EC_{50}), quantified before vaccination and two weeks after second vaccination with either mRNA or Rabipur. Dotted line designates assay lower limit of detection (LLOD). n=18 biologically independent animals. All statistical tests comparing the study groups were two-tailed tests.

a

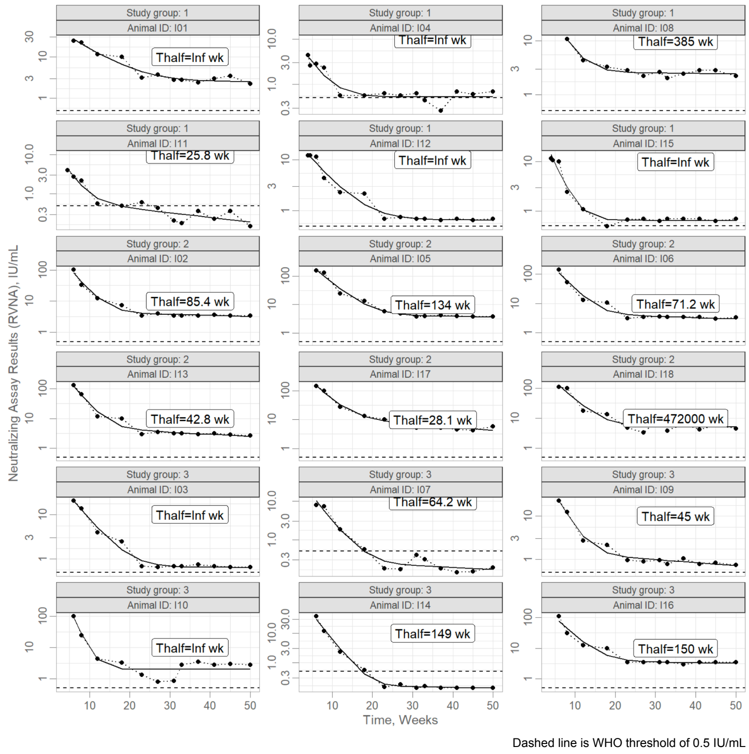

b

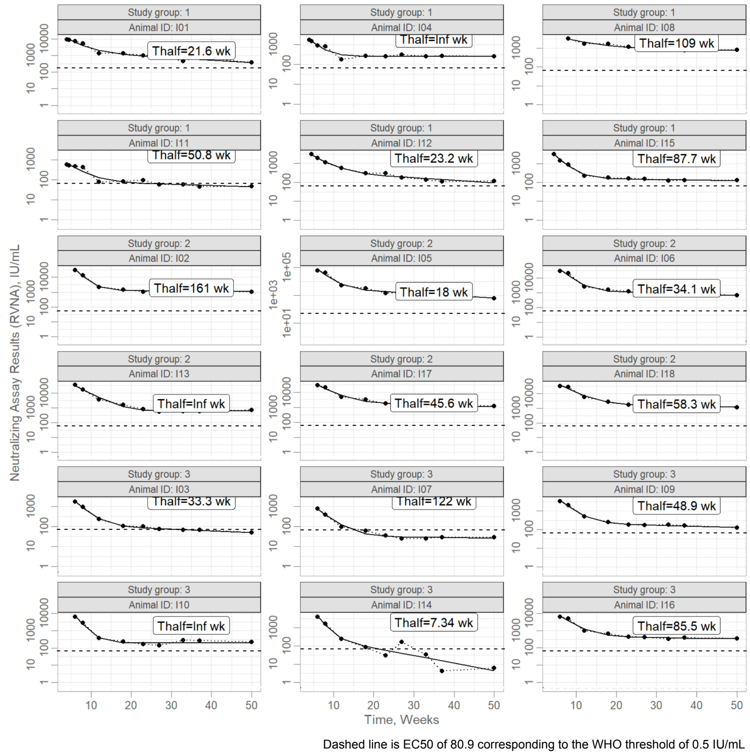

Figure S8

Supplementary Fig. 8: More durable half-life of antibodies by prime-boost mRNA vaccination. Half-life estimates for **(a)** neutralizing antibody titers and **(b)** total binding antibody titers are shown individually for each animal. Data lower than the maximum concentration value (Cmax) for each animal was censored, and time-after-Cmax (TAM=Week-Tmax) was computed. A two-compartment model was fit to TAM and concentration data. The terminal half-life is derived from these model estimates.

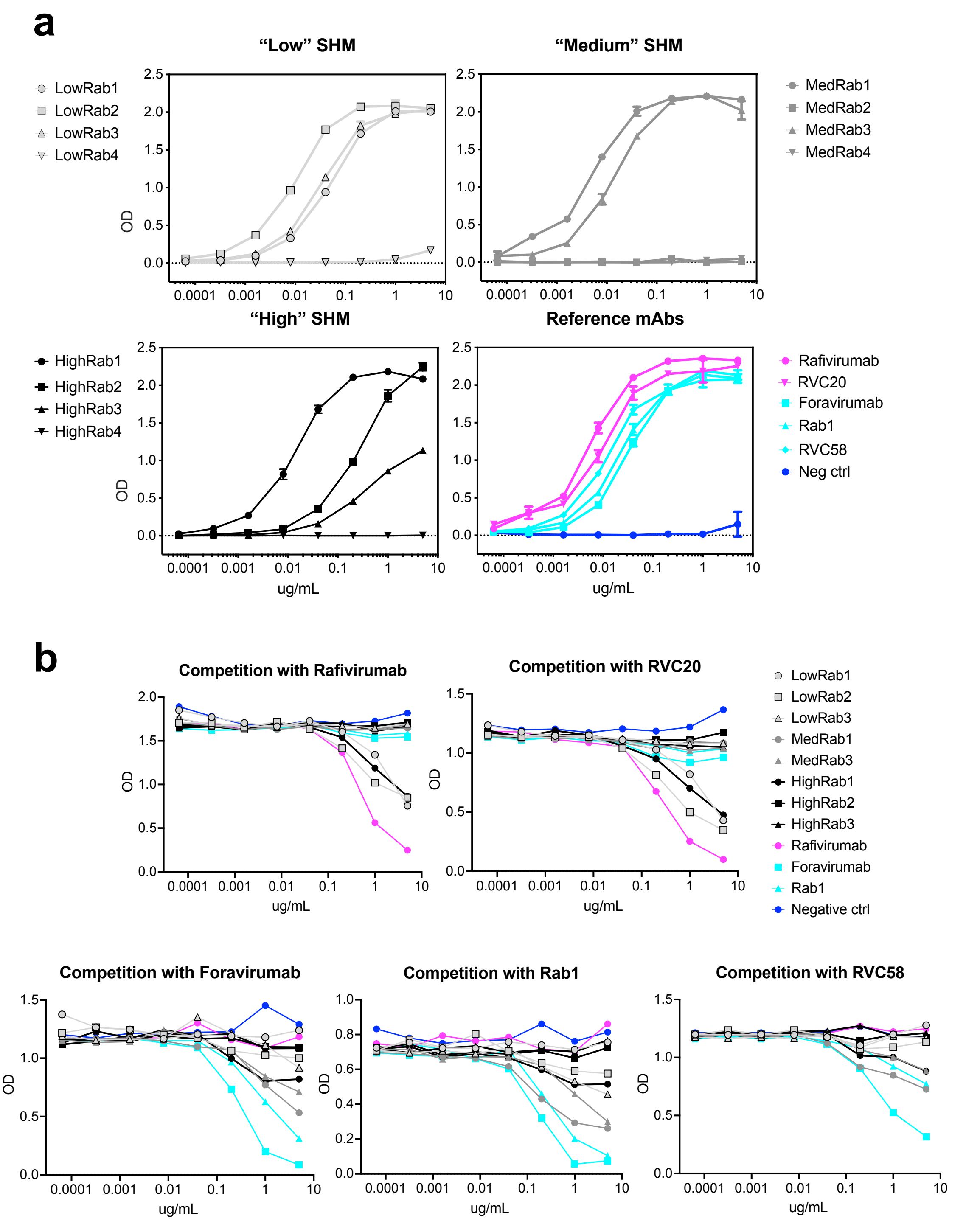

Figure S9

Supplementary Fig. 9: Binding and competition curves for the cloned monoclonal antibodies and for the therapeutic/reference rabies antibodies. (a) The full dilution series of the cloned monoclonal antibodies are shown separately for those with low SMH (green), medium SHM (yellow), high SHM (red) and for the therapeutic/reference rabies antibodies (Magenta and cyan for those binding to Site I and Site III respectively). Negative control is shown in blue. n=18 biologically independent animals. **(b)** The full dilution series of the cloned monoclonal antibodies are shown separately for competition with each of the therapeutic/reference rabies antibodies. Color coding is the same as in panel A. Error bars indicate geometric mean ± geometric SD.

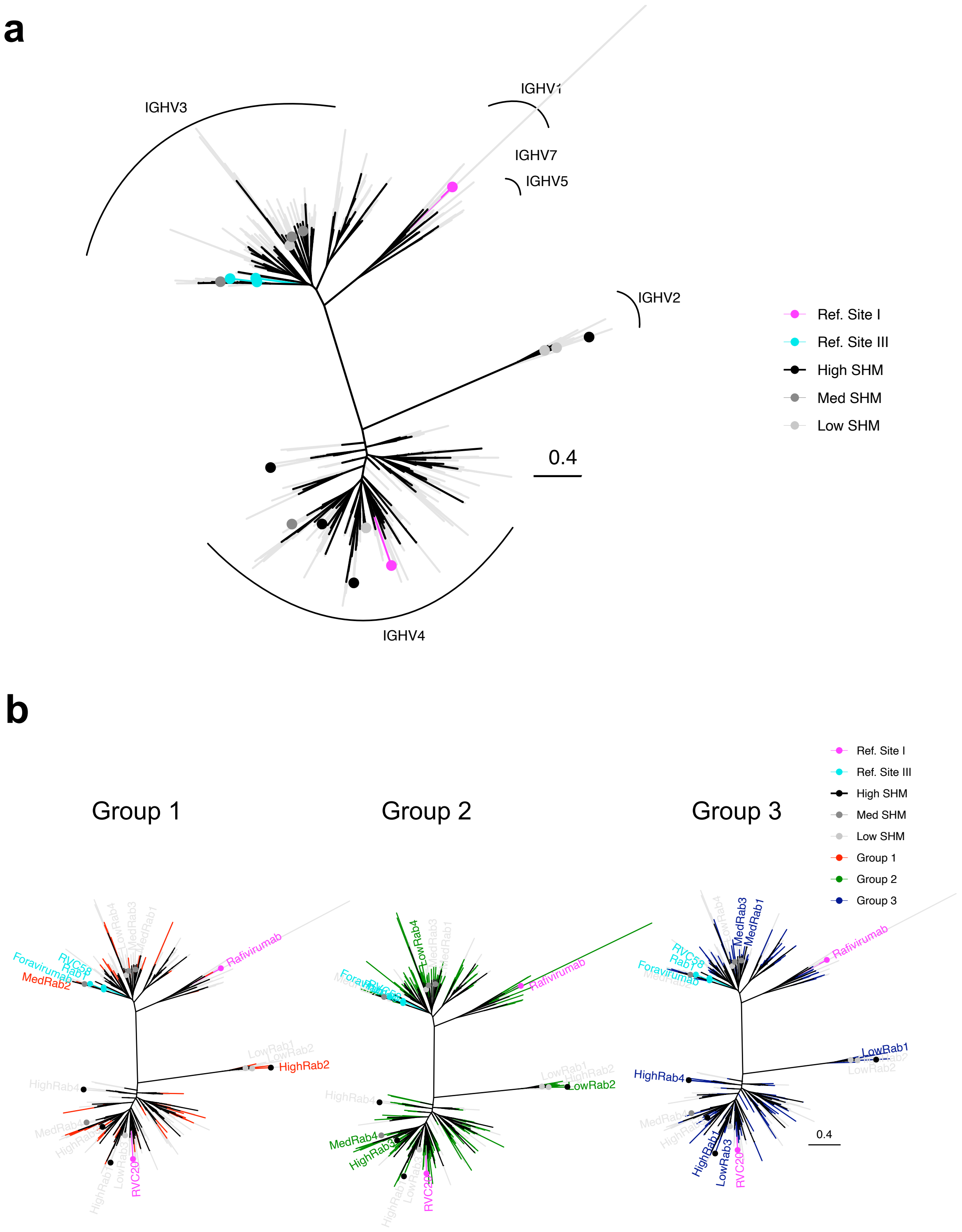

Figure S10

Supplementary Fig. 10: Overall high resemblance of the antibody sequences induced by the vaccines. Maximum likelihood phylogenetic tree generated by the alignment of VDJH amino acid sequences from all groups. The scale bar indicates the distance of 0.4 substitutions per sequence position. The sequence position of the therapeutic/reference rabies antibodies binding to Site I and to Site III is indicated by the magenta and cyan circles respectively along with the sequence position for the cloned mAbs (LowRab, MedRab and HighRab) indicated with light grey, dark grey and black circles respectively instead. The branches highlighted in black indicate those in common between the different groups. IGHV family branch annotations are included. **(b)** Maximum likelihood phylogenetic trees generated by the alignment of VDJH amino acid sequences are shown separately for the different groups. The scale bar indicates the distance of 0.4 substitutions per sequence position. The sequence position of the therapeutic/reference rabies antibodies binding to Site I and to Site III is indicated by the magenta and cyan circles respectively in each tree along with the sequence position for the cloned mAbs (LowRab, MedRab and HighRab) indicated with light grey, dark grey and black circles respectively instead. The branches highlighted in red, green and blue indicate those specific for each group.

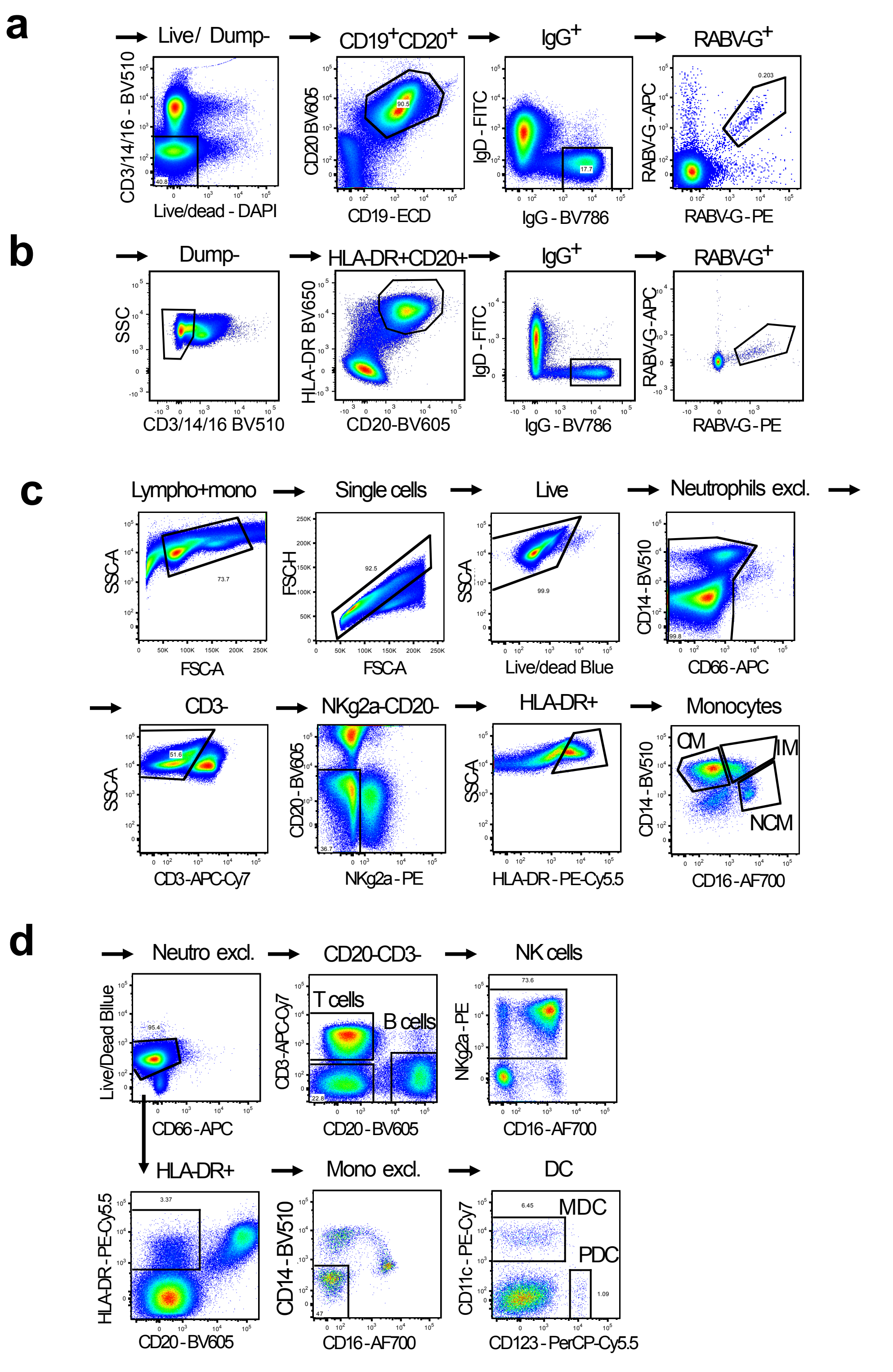

Figure S11

Supplementary Fig. 11: Flow cytometry gating for the identification of different lymphocyte subsets and of RABV-G+ memory B cells. (a) RABV-G+ memory B cells are identified as live single cells being CD3/14/16$^-$CD19$^+$CD20$^+$IgD$^-$IgG$^+$ and double positive for the RABV-G probe conjugated with 2 different fluorochromes. **(b)** Alternate identification of RABV-G+ memory B cells used for certain B cell sorts, identified as CD3/14/16$^-$HLA-DR$^+$CD20$^+$IgD$^-$IgG$^+$ and double positive for the RABV-G probe conjugated with 2 different fluorochromes. **(c)** Identification of monocyte subsets. Total monocytes are first identified as live single cells being CD66$^-$CD3$^-$Nkg2a$^-$CD20$^-$HLA-DR$^+$. Within this population, classical monocytes (CM) are then identified as CD14$^+$CD16$^-$, intermediate monocytes (IM) as CD14$^+$CD16$^+$ and non-classical monocytes (NCM) as CD14$^-$CD16$^+$. **(d)** Identification of other lymphocyte subsets. Live single cells being CD66$^-$ are first identified. Within this population, T cells are identified as CD3$^+$CD20$^-$, B cells as CD20$^+$CD3$^-$ and NK cells as CD3$^-$CD20$^-$NKg2a$^+$CD16$^{+/-}$. Total dendritic (DC) cells are instead first identified as HLA-DR$^+$CD20$^-$CD14$^-$CD16$^-$ with the myeloid DC (MDC) being CD11c$^+$CD123$^-$ and the plasmacytoid DC (PDC) being CD11c$^-$CD123$^+$.

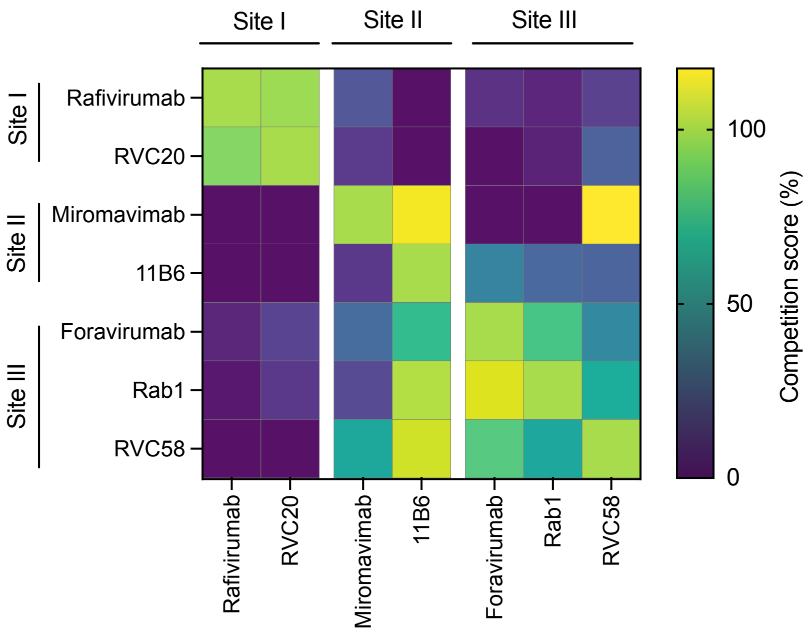

Figure S12

Supplementary Fig. 12: Cross-competition between RABVG-specific reference antibodies. Each reference mAb was tested in competition with the full reference panel and the percentage of cross-competition calculated using the area under the curve as described in Methods. Cross-competition percentages are displayed as heatmap.

Supplementary tables:

Supplementary Table 1. Simulation of antibody decay over time

Time to target* (weeks)	Group 1 (N=6)	Group 2 (n=6)	Group 3 (n=6)	Overall (n=18)
**Mean (SD)**	136 (155)	190 (77.0)	73.4 (89.6)	129 (117)
**Median (Min, Max)**	98.9 (17.9, 407)	188 (105, 279)	25.9 (13.8, 226)	101 (13.8, 407)
**Missing**	1 (16.7%)	2 (33.3%)	1 (16.7%)	4 (22.2%)

*A binding assay target of 80.9 EC50 was set, corresponding to the neutralizing assay target of 0.5 IU/mL. Simulations out to ten years were performed, using the model fits for each animal. The time to binding assay target was calculated for each animal, and summaries per group are reported.

Supplementary Table 2. Flow cytometry panels

a. Immunophenotyping of innate leukocyte subsets

Fluorochrome	Marker	Company	Cat. No.	Clone	uL/test
**PerCP-Cy5.5**	CD123	BD	558714	7G3	3
**FITC**	CD40	BioLegend	334306	5C3	3
**PE-Cy7**	CD11c	BioLegend	301618	3.9	3
**Pe-Cy5.5**	HLA-DR	Invitrogen	MHLDR18	TU36	1
**PE-CF594**	CD70	BD	562484	Ki-24	3
**PE**	NKg2a (CD159a)	Beckman Coulter	Z199	IM3291U	2
**APC-Cy7**	CD3	BD	557757	SP34-2	2
**Alexa Fluor 700**	CD16	BD	560713	38G	1
**APC**	CD66	Miltenyi	130-093-155	TET2	2
**BV605**	CD20	BioLegend	302334	2H7	2
**BV510**	CD14	BioLegend	301842	M5E2	1
**BV421**	CD80	Biolegend	305222	2D10	1
**(DAPI)**	Live/Dead blue	ThermoFisher	L-23105		0.125

b. Staining of circulating antigen-specific memory B cells (1)

Fluorochrome	Marker	Company	Cat. No.	Clone	uL/test
**PerCP-Cy5.5**	IgM	BD	561285	G20-127	2.5
**FITC**	IgD	Southern Biotech	2030-02	Polyclonal	0.6
	7AAD	Thermofisher	A1210		0.025
**ECD**	CD19	Beckman Coulter	IM2708U	J3-119	4
**PE**	RABV-G				
**APC**	RABV-G				
**BV786**	IgG	BD	564230	G18-145	1.25
**BV605**	CD20	BioLegend	302334	2H7	0.3
**BV510**	CD3	BD	740187	SP34-2	2.5
**BV510**	CD14	BioLegend	301842	M5E2	0.6
**BV510**	CD16	BD	563830	3G8	1.25

c. Staining of circulating antigen-specific memory B cells (2)

Fluorochrome	Marker	Company	Cat. No.	Clone	uL/test
**PerCP-Cy5.5**	IgM	BD	561285	G20-127	2.5
**FITC**	IgD	Southern Biotech	2030-02	Polyclonal	0.6
	7AAD	Thermofisher	A1210		0.025
**ECD**	CD19	Beckman Coulter	IM2708U	J3-119	4
**PE**	RABV-G				
**APC**	RABV-G				
**BV786**	IgG	BD	564230	G18-145	1.25
**BV605**	CD20	BioLegend	302334	2H7	1
**BV510**	CD3	BD	740187	SP34-2	2.5
**BV510**	CD14	BioLegend	301842	M5E2	0.6
**BV510**	CD16	BD	563830	3G8	1.25
**BV650**	HLA-DR	Biolegend	307650	L243	1.25

d. Staining of circulating antigen-specific memory T cells

Fluorochrome	Marker	Company	Cat. No.	Clone	uL/test
**AF488**	TNF*	BD	560493	MAB11	3
**PE-Cy5.5**	CD4	Thermofisher	MHCD0418	S3.5	1.25
**PE-Cy5**	CD45RA	BD	552888	5H9	0.5
**ECD**	CD69*	Beckman Coulter	6607110	TP1.55.3	1.5
**PE**	IL-13*	BD	560493	JES10-5A2	3
**APC-Cy7**	CD3*	BD	557757	SP34-2	1.25
**AF700**	IFNg*	BioLegend	506516	B27	0.5
**AF647**	IL-21*	BD	560493	3A3-N2.1	5

BV786	IL-17A*	BioLegend	512338	BL168	1.5
BV605	IL-2*	BD	559334	MQ1-17H12	2
**BV570**	CD8	Biolegend	301038	RPA-T8	0.625
	Live/Dead Aqua	Thermofisher	L34957		0.3
**BV421**	CCR7	Biolegend	353208	G043H7	2

* Intracellular staining

Supplementary Table 3. Sequencing read statistics

ID	Single cell sequences	Single cell clonotype counts	HTS lineage tracing	HTS raw read counts	HTS final sequences after QC filtering
I01	7	7	0	2321921	1168419
I02	50	48	38	1794649	873331
I03	38	22	1	1077087	665425
I05	75	60	205	2374279	1047994
I06	68	65	19	1693502	864606
I07	40	29	24	1716898	800813
I08	17	17	0	2908401	1136492
I09	65	48	101	1391230	766161
I10	70	47	80	1869105	894740
I11	1	1	0	1729383	834352
I12	23	22	1	1592507	711002
I13	11	11	0	2477894	1415348
I14	58	45	29	2034000	951335
I15	19	19	0	1467541	746862
I16	243	81	358	2636283	1265542
I17	199	176	132	2050913	1038466
I18	6	6	0	2147462	1064503
