# Supplementary material for: Unmodified rabies mRNA vaccine elicits high cross-neutralizing antibody titers and diverse B cell memory responses
Source: Nat Commun. 2023 Jun 22;14:3713. doi: 10.1038/s41467-023-39421-5 (PMC10287699; doi:10.1038/s41467-023-39421-5)
Supplement: Supplementary file 3 — Reporting Summary [file 41467_2023_39421_MOESM3_ESM.pdf]

nature portfolio

Corresponding author(s): Karin Loré

Last updated by author(s): Feb 10, 2023

Reporting Summary

Nature Portfolio wishes to improve the reproducibility of the work that we publish. This form provides structure for consistency and transparency in reporting. For further information on Nature Portfolio policies, see our Editorial Policies and the Editorial Policy Checklist.

Statistics

For all statistical analyses, confirm that the following items are present in the figure legend, table legend, main text, or Methods section.

n/a	Confirmed	
☐	☒	The exact sample size (*n*) for each experimental group/condition, given as a discrete number and unit of measurement
☐	☒	A statement on whether measurements were taken from distinct samples or whether the same sample was measured repeatedly
☐	☒	The statistical test(s) used AND whether they are one- or two-sided<br>*Only common tests should be described solely by name; describe more complex techniques in the Methods section.*
☐	☒	A description of all covariates tested
☐	☒	A description of any assumptions or corrections, such as tests of normality and adjustment for multiple comparisons
☐	☒	A full description of the statistical parameters including central tendency (e.g. means) or other basic estimates (e.g. regression coefficient) AND variation (e.g. standard deviation) or associated estimates of uncertainty (e.g. confidence intervals)
☐	☒	For null hypothesis testing, the test statistic (e.g. *F*, *t*, *r*) with confidence intervals, effect sizes, degrees of freedom and *P* value noted<br>*Give P values as exact values whenever suitable.*
☒	☐	For Bayesian analysis, information on the choice of priors and Markov chain Monte Carlo settings
☒	☐	For hierarchical and complex designs, identification of the appropriate level for tests and full reporting of outcomes
☒	☐	Estimates of effect sizes (e.g. Cohen's *d*, Pearson's *r*), indicating how they were calculated

Our web collection on statistics for biologists contains articles on many of the points above.

Software and code

Policy information about availability of computer code

Data collection	Flow cytometry data was collected using commercially available BD FACSDiva software(v8.1 or 9.1, Beckton Dickinson). ELISA data was partially collected using commercially available SkanIt software (v 7.0, Thermo Fisher). Luminex data was collected using commercially available Bioplex Manager Software (v 6.2, Bio-Rad).
Data analysis	B cell receptor sequences were processed using IgDiscover software (v 0.15.1), sequences were aligned using MUSCLE (v 5.1), and phylogenetic trees were generated using FastTree (2.1.11). The 3D model of trimeric RABV-G was edited and colored using UCSD Chimera X (v 1.2.5). Custom code and all R packages and its dependencies together with their respective versions used for data analysis are publicly available on GitHub repository under an R environment lock file (github.com/rodrigarc/rabies_mRNA). Flow data analysis was performed using Flowjo (v 9.0, Treestar) . Statistical analyses were performed using Graphpad Prism version 9.0 (La Jolla California, USA) or R (v 4.2.2.) for Mac OS X.

For manuscripts utilizing custom algorithms or software that are central to the research but not yet described in published literature, software must be made available to editors and reviewers. We strongly encourage code deposition in a community repository (e.g. GitHub). See the Nature Portfolio guidelines for submitting code & software for further information.

Data

Policy information about availability of data

All manuscripts must include a data availability statement. This statement should provide the following information, where applicable:

- Accession codes, unique identifiers, or web links for publicly available datasets
- A description of any restrictions on data availability
- For clinical datasets or third party data, please ensure that the statement adheres to our policy

All the sequencing data generated in this study are available in the NCBI database under a Bioproject [PRJNA932987]. Processed sequences following the AIRR-standards are available at Zenodo repository with the acession number 7680334. The KIMDB database was used to retrieve the Macaca mulatta germline sequences (Available at: http://kimdb.gkhlab.se/datasets/). The B cell receptor sequences that were expressed as monoclonal antibodies are available as supplemental material.

Human research participants

Policy information about studies involving human research participants and Sex and Gender in Research.

Reporting on sex and gender	N/A
Population characteristics	N/A
Recruitment	N/A
Ethics oversight	N/A

Note that full information on the approval of the study protocol must also be provided in the manuscript.

Field-specific reporting

Please select the one below that is the best fit for your research. If you are not sure, read the appropriate sections before making your selection.

☒ Life sciences ☐ Behavioural & social sciences ☐ Ecological, evolutionary & environmental sciences

For a reference copy of the document with all sections, see nature.com/documents/nr-reporting-summary-flat.pdf

Life sciences study design

All studies must disclose on these points even when the disclosure is negative.

Sample size	The number of non-human primates used was based on the number of animals available and standard practice in the field.
Data exclusions	One animal was not sampled at week 4 and 4.5 due to health status and data from this animal is thereby excluded from analysis where appropriate. Data points deemed unreliable due to technical experimental issues were excluded in few cases.
Replication	Due to limitations in large animal trials, non-human primate immunization experiments were not replicated. All the samples were run as technical duplicates when possible, this includes all the ELISAs and the neutralization assays performed in this study.
Randomization	Animals were not randomly assigned to the groups. The vaccination grouping was done controlling for sex and weight in order to have similar distribution of these factors between the study groups.
Blinding	Investigators were not blinded to data collection and/or data analysis due to lack of personnel and resources.

Reporting for specific materials, systems and methods

We require information from authors about some types of materials, experimental systems and methods used in many studies. Here, indicate whether each material, system or method listed is relevant to your study. If you are not sure if a list item applies to your research, read the appropriate section before selecting a response.

Materials & experimental systems

n/a	Involved in the study
☐	☒ Antibodies
☒	☐ Eukaryotic cell lines
☒	☐ Palaeontology and archaeology
☐	☒ Animals and other organisms
☒	☐ Clinical data
☒	☐ Dual use research of concern

Methods

n/a	Involved in the study
☒	☐ ChIP-seq
☐	☒ Flow cytometry
☒	☐ MRI-based neuroimaging

Antibodies

Antibodies used	Information about all the antibodies used in the flow cytometry pannels are described in supplementary tables (Table S2). The antibodies used for ELISAs are described in their corresponding method's section.
Validation	Reactivity of all antibody reagents used in this study were based on manufacturer's reported information on manufacturer's website.

Animals and other research organisms

Policy information about studies involving animals; ARRIVE guidelines recommended for reporting animal research, and Sex and Gender in Research

Laboratory animals	Eighteen Chinese rhesus macaques (Macaca mulatta) of approximate 3.5 years of age were used in this study.
Wild animals	This study did not involve wild animals.
Reporting on sex	Eighteen Chinese rhesus macaques (Macaca mulatta), nine males and nine females, were used in this study. Animals were divided into three study groups keeping similar distribution of sex and body weight.
Field-collected samples	This study did not involve field-collected samples.
Ethics oversight	This study was approved by the Stockholm Regional Ethical Board on Animal Experiments (18427-2019).

Note that full information on the approval of the study protocol must also be provided in the manuscript.

Flow Cytometry

Plots

Confirm that:

☒ The axis labels state the marker and fluorochrome used (e.g. CD4-FITC).

☒ The axis scales are clearly visible. Include numbers along axes only for bottom left plot of group (a 'group' is an analysis of identical markers).

☒ All plots are contour plots with outliers or pseudocolor plots.

☒ A numerical value for number of cells or percentage (with statistics) is provided.

Methodology

Sample preparation	PBMC were isolated using a ficoll gradient protocol and cryopreserved until use. For analysis of innate immune cell populations and plasmablasts, PBMCs were stained with fluorescently labeled antibodies directly after isolation. For other assays, PBMCs were thawed in batches and stained with a panel of fluorescently labelled antibodies to identify memory B cells. For T cell assays, thawed PBMC were stimulated with antigenic peptides as described in the methods section and then stained with a panel of fluorescent antibodies for analysis of intracellular cytokines.
Instrument	All samples, with the exception of week 18, were acquired on a BD LSRFortessa flow cytometer. Week 18 samples were acquired on a BD FACSAria Fusion instrument in order to combine data acquisition with simultaneous single-cell sorting into 96-well PCR plates. Additional sorting of antigen-specific cells from week 8 for BCR sequencing was also performed on a BD FACSAria Fusion instrument.
Software	Data was acquired with BD FACSDiva (Beckton Dickinson) version 8.0.1 or 9.0.1, and analyzed using Flowjo versions 9 or 10 (BD Life Sciences).
Cell population abundance	Single-cell index sorts were performed for the isolation of RABVG-specific memory B cells.
Gating strategy	Details regarding the gating strategy for each of the subset studied is described in detail in the supplementary material with representative figures. Total CD4/CD8+ memory T cells are first identified as live single cells being CD3+CD8-CD45RA-CCR7+/-

for CD4 memory and CD3+CD8+CD45RA-CCR7+/- for CD8 memory. Within this population, activated cells being CD69+ are analyzed for the presence of different intracellular cytokines; IFNg, IL-2, IL-17A, IL-13 and IL-21. RABV-G+ memory B cells are identified as live single cells being CD3/14/16-CD19+CD20+IgD-IgG+ and double positive for the RABV-G probe. For week 8 sorts a slightly alternate identification of RABV-G+ memory B cells was used, identifying RABVG+ memory B cells as CD3/14/16-HLA-DR+CD20+IgD-IgG+ and double positive for the RABV-G probe conjugated with 2 different fluorochromes. Monocytes are first identified as live single cells being CD66-CD3-Nkg2a-CD20-HLA-DR+. Within this population, classical monocytes (CM) are then identified as CD14+CD16-, intermediate monocytes (IM) as CD14+CD16+ and non-classical monocytes (NCM) as CD14-CD16+. For identification of lymphocyte and dendritic cell subsets, live single cells being CD66- are first identified. Within this population, T cells are identified as CD3+CD20-, B cells as CD20+CD3- and NK cells as CD3-CD20-NKg2a+CD16+/-. Total dendritic (DC) cells are instead first identified as HLA-DR+CD20-CD14-CD16- with the myeloid DC (MDC) being CD11c+CD123- and the plasmacytoid DC (PDC) being CD11c-CD123+.

☒ Tick this box to confirm that a figure exemplifying the gating strategy is provided in the Supplementary Information.
